# Development and External Validation of the STRATified CANcer Surveillance (STRATCANS) Multivariable Model for Predicting Progression in Men with Newly Diagnosed Prostate Cancer Starting Active Surveillance

**DOI:** 10.3390/jcm12010216

**Published:** 2022-12-27

**Authors:** Alexander Light, Artitaya Lophatananon, Alexandra Keates, Vineetha Thankappannair, Tristan Barrett, Jose Dominguez-Escrig, Jose Rubio-Briones, Toufik Benheddi, Jonathan Olivier, Arnauld Villers, Kirthana Babureddy, Haitham Abdelmoteleb, Vincent J. Gnanapragasam

**Affiliations:** 1 Division of Urology, Department of Surgery, University of Cambridge, Cambridge CB2 0QQ, UK; 2 Department of Urology, Cambridge University Hospitals NHS Foundation Trust, Cambridge CB2 0QQ, UK; 3 Cambridge Urology Translational Research and Clinical Trials Office, Cambridge Biomedical Campus, University of Cambridge, Cambridge CB2 0QQ, UK; 4 Division of Population Health, Health Services Research and Primary Care, University of Manchester, Manchester M13 9PL, UK; 5 Department of Radiology, University of Cambridge, Cambridge CB2 0QQ, UK; 6 Department of Radiology, Cambridge University Hospitals NHS Foundation Trust, Cambridge CB2 0QQ, UK; 7 Department of Urology, Fundación Instituto Valenciano de Oncología, 46009 Valencia, Spain; 8 Department of Urology, Lille University, 59000 Lille, France; 9 UMR8161, CNRS-Institut de Biologie de Lille, 59800 Lille, France; 10 Department of Urology, University Hospital of Wales, Cardiff and Vale University Health Board, Cardiff CF14 4XW, UK

**Keywords:** prostate cancer, active surveillance, Cambridge Prognostic Groups (CPG), risk prediction, risk model, non-metastatic disease, MRI, PSA, biopsy

## Abstract

For men with newly diagnosed prostate cancer, we aimed to develop and validate a model to predict the risk of progression on active surveillance (AS), which could inform more personalised AS strategies. In total, 883 men from 3 European centres were used for model development and internal validation, and 151 men from a fourth European centre were used for external validation. Men with Cambridge Prognostic Group (CPG) 1–2 disease at diagnosis were eligible. The endpoint was progression to the composite endpoint of CPG3 disease or worse (≥CPG3). Model performance at 4 years was evaluated through discrimination (C-index), calibration plots, and decision curve analysis. The final multivariable model incorporated prostate-specific antigen (PSA), Grade Group, magnetic resonance imaging (MRI) score (Prostate Imaging Reporting & Data System (PI-RADS) or Likert), and prostate volume. Calibration and discrimination were good in both internal validation (C-index 0.742, 95% CI 0.694–0.793) and external validation (C-index 0.845, 95% CI 0.712–0.958). In decision curve analysis, the model offered net benefit compared to a ‘follow-all’ strategy at risk thresholds of ≥0.08 and ≥0.04 in development and external validation, respectively. In conclusion, our model demonstrated good accuracy and clinical utility in predicting the progression on AS at 4 years post-diagnosis. Men with lower risk predictions could subsequently be offered less-intense surveillance. Further external validation in larger cohorts is now required.

## 1. Introduction

Active surveillance (AS) favourable-risk prostate cancer is increasingly popular [1]. Modern AS strategies include serial prostate-specific antigen (PSA) measurements, MRIs, and biopsies to identify disease progression necessitating treatment. However, the optimal investigation frequency remains unclear. Furthermore, there is disagreement regarding eligibility criteria and discontinuation triggers [2]. Accordingly, guidelines and practice vary widely [3,4,5,6,7]. In the UK, national guidelines recommend active surveillance as a management option for men with Cambridge Prognostic Group (CPG) 1 or 2 disease (Table 1) [3]. An accompanying protocol recommends PSA should be measured every 3–4 months in the first year, then every 6 months; digital rectal examination should be performed every 12 months and a multiparametric MRI at 12–18 months.

The optimal surveillance regime is unlikely to be a one-size-fits-all strategy given the biological heterogeneity of localised disease. Furthermore, repeat biopsies risk complications such as sepsis and urinary retention, and these should be minimised as far as safe and practical [8]. Third, compliance is generally poor with uniform strategies, particularly following repeat biopsies [9,10,11,12]. Ideally, an accurate model for predicting progression could underpin more individualised AS strategies, improving adherence and reducing investigations, but without missing progression.

The definition of progression is also vital. We previously proposed a pragmatic endpoint of progression to CPG3 or higher (≥CPG3) (Table 1) [13,14,15]. CPG1 and 2 represent low and favourable-intermediate risk groups, respectively, and these men do not gain significant survival benefit with treatment [15]. Therefore, they could be good AS candidates. Conversely, CPG3 disease (unfavourable-intermediate risk) that is untreated is associated with significantly higher mortality. Consequently, we argue ≥CPG3 progression represents a strong trigger for ending AS and commencing treatment.

Here, using biochemical, radiological, and pathological data from a multicentre cohort of men starting AS, we develop and validate a multivariable model for predicting ≥CPG3 progression.

## 2. Materials and Methods

### 2.1. Study Population

The study population comprised men enrolled in 4 AS programmes with prospectively-maintained databases starting from at least 2013. To ensure powered model development, we used a multicentre cohort from the UK (Cambridge), France (Lille), and Spain (Valencia). For external validation, we used a separate single-centre cohort from Cardiff, UK.

At the study level, men included were CPG1–2 at diagnosis (Table 1). Accordingly, all were stage T1–2, with PSA < 20 ng/mL if grade group (GG) 1, or PSA ≤ 10 ng/mL if GG2. We excluded men with ≥CPG3 disease at diagnosis, men without a histological diagnosis, and men with insufficient baseline data to determine CPG, or at least 1 surveillance investigation result.

### 2.2. Development Cohorts

**Figure 1:**
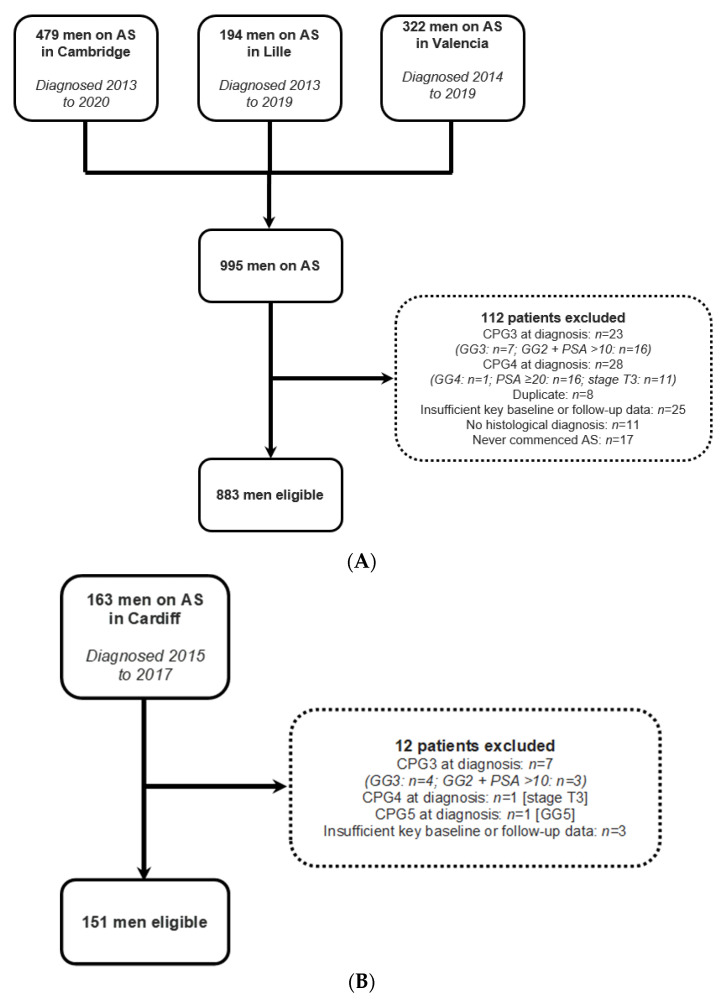
Data assembly process for the (**A**) development cohorts, and (**B**) external validation cohort. AS, active surveillance; CPG, Cambridge Prognostic Group; GG, grade group.

Across the 3 cohorts, the median time-to-progression was 1.7 years (interquartile range (IQR) 0.8–3.1), and the median follow-up of censored men was 3.0 years (IQR 1.5–4.7) (Figure S1). Centre-specific data are shown in Table 2, Figure 1 and Figure S1. Further details regarding local AS eligibility criteria and surveillance protocols are given in Supplementary Text S1.

### 2.3. Outcome

Our predicted AS endpoint was ≥CPG3 progression (Table 1). Meeting this composite endpoint was possible through 4 scenarios: (i) upgrading to ≥GG3 disease on repeat biopsy; (ii) GG2 disease (on initial or repeat biopsy) with PSA ≥ 10 ng/mL during surveillance; (iii) radiological progression to T3–4 disease; (iv) PSA > 20 ng/mL during surveillance. For PSA-related progression, 2 consecutive PSA values in the target range were needed. Men who did not progress were censored at the date of treatment, death, or latest investigation (PSA, MRI, biopsy), whichever occurred first. Neither clinicians nor the study team were blinded to baseline or outcome data.

### 2.4. Statistical Analyses

All analyses were performed using R v.3.6.1. Statistical significance was denoted by *p* < 0.05.

We considered the following baseline variables for model development: PSA, GG, age, family history, prostate volume, MRI score (between 1 and 5, using the Prostate Imaging-Reporting and Data System (PI-RADS v1 or v2, dependent on use at time) or Likert measures), and core positivity (number of positive biopsy cores divided by total cores). Cambridge utilised the Likert system, whilst the other centres used PI-RADS. PSA, prostate volume, MRI score and core positivity were modelled as continuous variables. Missing data for MRI score, prostate volume, and core positivity were derived using multiple imputations by chained equations with 30 iterations and 30 imputations using the *mice* R package.

Multivariable Cox proportional hazards regression was used to model ≥CPG3 progression in our development cohort. All variables were considered and then sequentially removed via backward selection if non-significant. Elimination stopped once all remaining variables were statistically significant. The proportional hazards assumption was tested by plotting Schoenfeld residuals against time. For the final model, we confirmed adequate power regarding the number of participants, events, and predictor parameters through three criteria: (i) small optimism in the predictor effect defined by a global shrinkage factor ≥0.9; (ii) small absolute difference of ≤0.05 in the model’s apparent and adjusted Nagelkerke’s *R*^2^; (iii) precise estimation of the overall risk in the population, defined as a stringent margin of error ≤0.05 in outcome proportion estimates for a null model [16].

For the final model, we calculated adjusted hazard ratios (HR) with 95% confidence intervals (CI). We then internally validated the model using bootstrapping with 10,000 repetitions. We externally validated the model in the Cardiff cohort by calculating the linear predictor using model coefficients. Model performance was assessed at 4 years post-diagnosis. 43.3%, 36.0%, 20.0%, and 44.0% of censored men had at least 4 years of follow-up in the Cambridge, Lille, Valencia, and Cardiff cohorts, respectively (Figure S1).

Prognostic accuracy was assessed using the concordance index (C-index). We evaluated model calibration graphically by plotting predicted vs. observed progression-free survival, and through calculation of the calibration slope. To assess clinical utility, we performed decision curve analysis [17]. Model-based decision making was compared against strategies of ‘follow-all’ (one-size-fits-all surveillance) and ‘follow-none’ surveillance strategies. Net benefit was estimated by summing benefits (detecting ≥CPG3 progression) and subtracting harms (further biopsies, MRIs, and PSA measurements). Net benefit was calculated for a range of risk thresholds, representing how many men a clinician would investigate to detect 1 progression event. If a clinician is willing to investigate 10 men to detect 1 progression event, this implies detecting 1 progression event is worth 9 investigations that do not. Here, the risk threshold is 0.11 (1 divided by 9). The most clinically useful model at a given risk threshold will have the greatest net benefit. We also calculated the net reduction in investigations per 100 men, adjusted for false negative investigations. This indicates the reduction in investigations without missing a progression event.

### 2.5. Sensitivity Analyses

First, we excluded men with imputed data. Second, we excluded men diagnosed with GG1 cancer, but experienced reclassification to GG3 disease or worse within 1 year. This aimed to exclude men with inadequately-characterised disease who are more likely to exhibit reclassification than genuine progression [18,19].

## 3. Results

### 3.1. Development Cohort

Of the 995 men in the multicentre development cohort, 883 were included, with exclusion reasons given in Figure 1. Table 2 displays baseline characteristics. Missing data were imputed for family history in 94 men (10.6%), prostate volume in 12 (1.4%), MRI score in 161 (18.2%), and core positivity in 48 (5.4%).

**Figure 2:**
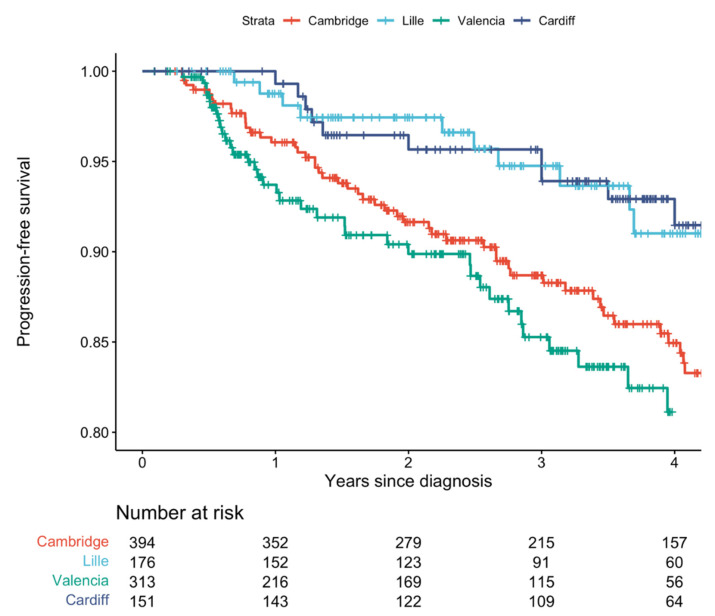
Kaplan–Meier curves for each centre’s cohort, with outcome being progression to Cambridge Prognostic Group 3 or worse. The multicentre model development cohort comprised men from Cambridge, Lille, and Valencia. The Cardiff cohort was used for external validation.

Overall, 106 men (12.0%) progressed to ≥CPG3. Of these, 46 (43.4%) had disease upgrading to ≥GG3, 15 (14.2%) had GG2 disease (on initial or repeat biopsy) with PSA ≥ 10 ng/mL during surveillance, 18 (17.0%) progressed radiologically to stage T3–4, and 28 (26.4%) reached PSA > 20 ng/mL during surveillance (Table S1). By 4 years, 93 men (10.5%) had reached the endpoint. No patient developed metastases or died of prostate cancer during the study follow-up. Figure 2 displays Kaplan–Meier curves for each cohort. 235 men (26.6%) discontinued AS for any reason, including treatment after identifying ≥CPG3 progression. Of the 777 men who did not reach ≥CPG3 progression, 168 (21.6%) discontinued AS during follow-up (Figure S2).

### 3.2. Model Development and Internal Validation

**Figure 3:**
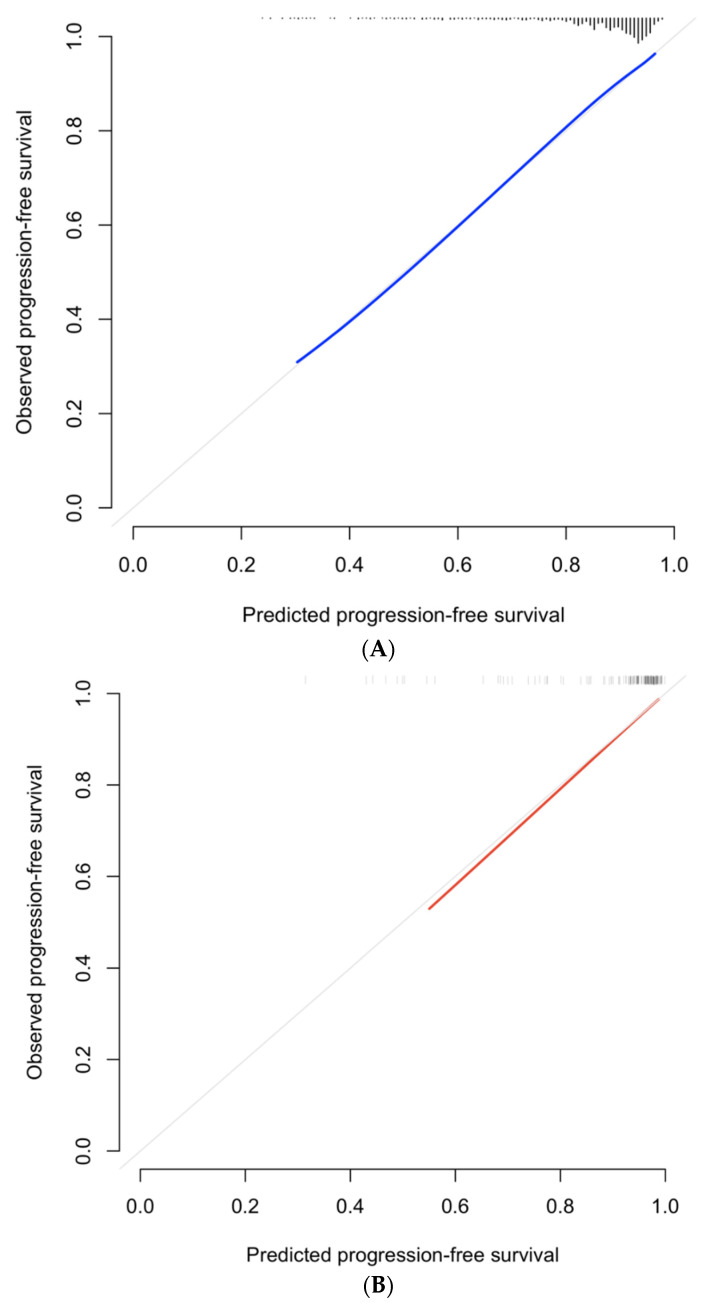
Calibration curves at 4 years for internal validation (**A**), and external validation (**B**).

**Figure 4:**
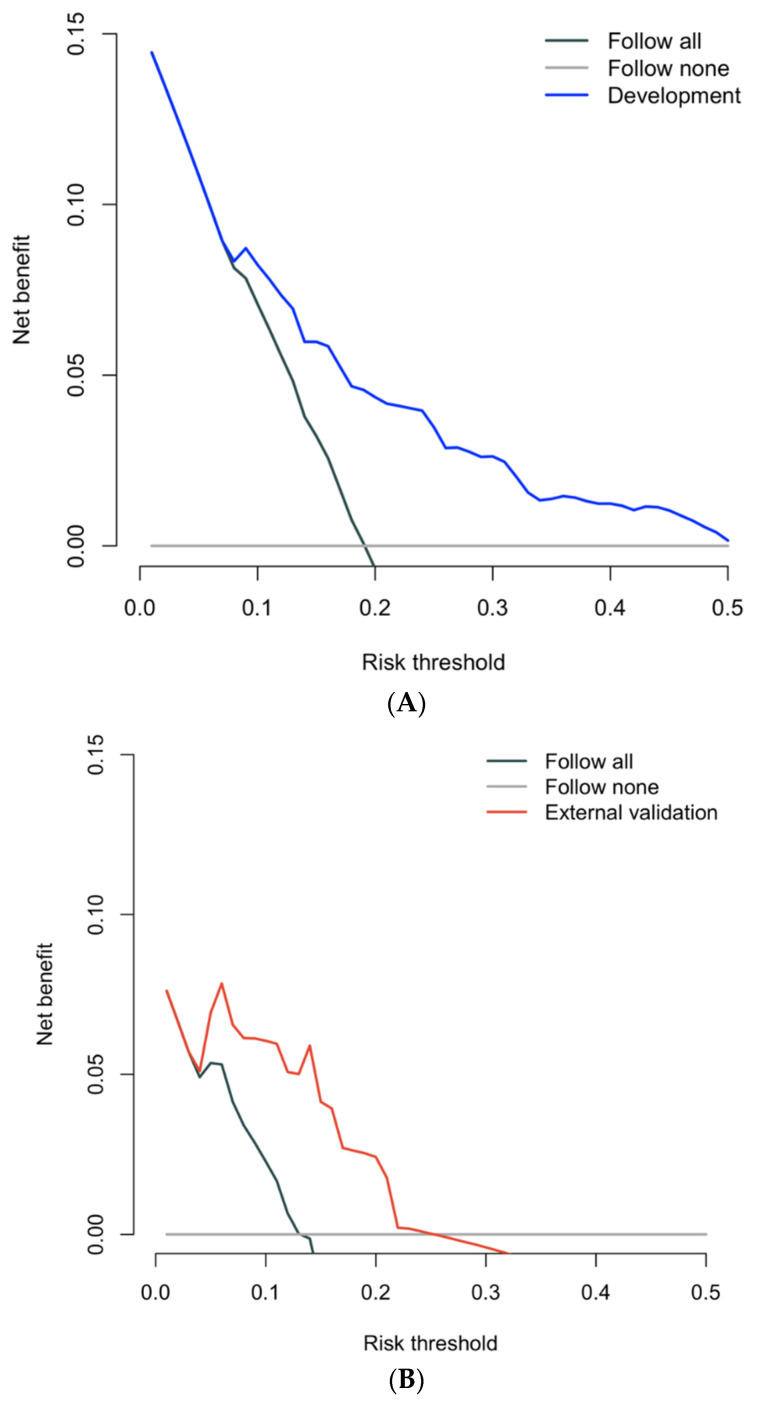
Decision curve analyses at 4 years in development (**A**) and external validation (**B**).

Unadjusted associations between predictors and outcomes are shown in Table S2. In the final multivariable model, baseline variables significantly associated with ≥CPG3 progression were PSA (HR 1.20, 95% CI 1.13–1.27, *p* < 0.001), GG2 (HR 4.16, 95% CI 2.64–6.54, *p* < 0.001), MRI score 4–5 (HR 1.85, 95% CI 1.21–2.84, *p* < 0.001), and prostate volume (HR 0.990, 95% CI 0.982–0.0.998, *p* = 0.01). Discrimination was good in both development (C-index 0.749, 95% CI 0.701–0.799) and internal validation (C-index 0.742, 95% CI 0.694–0.793). Calibration was also good. In internal validation, visual inspection of the calibration plot demonstrated close agreement between predicted and observed progression-free survival (Figure 3). Calibration slope was 0.982 in internal validation. In decision curve analysis, there was incremental net benefit compared to a strategy of ‘follow-all’ at risk thresholds ≥0.08 (Figure 4). This equates to net benefit for a clinician who would investigate at most 12.5 men to detect 1 progression event.

### 3.3. Sensitivity and Subgroup Analyses

We excluded 167 men with imputed data for the final variables. C-index was 0.743 (95% CI 0.687–0.808) in the development cohort and 0.743 (95% CI 0.689–0.817) in internal validation. Calibration remained good on inspection of calibration plots in internal validation, with calibration slope 1.007 (Figure S3). Next, we excluded 20 men with GG1 disease and upgraded to GG3 disease within 1 year. C-index was 0.812 (95% CI 0.758–0.851) in the development cohort, and 0.812 in internal validation (95% CI 0.757–0.854). The calibration plot showed some slight underestimation of progression-free survival for most risk predictions (Figure S4). Calibration slope was 1.003.

### 3.4. External Validation

163 men were enrolled in the Cardiff programme, of whom 151 were included (Figure 1, Table 2). Missing data were imputed for prostate volume in 2 (1.3%), MRI score in 6 (4.0%), and core positivity in 3 (2.0%). 11 men (7.3%) progressed to ≥CPG3. Of these, 3 (27.3%) had disease upgrading to ≥GG3 on repeat biopsy, and 8 (72.7%) had GG2 disease (on initial or repeat biopsy) with PSA ≥ 10 ng/mL during surveillance. 9 men (6.0%) reached the endpoint within 4 years. No patient developed metastatic disease or died of prostate cancer during the study follow-up.

Model discrimination was high (C-index 0.845, 95% CI 0.712–0.958). Furthermore, an inspection of the calibration plot demonstrated close agreement between predicted and observed progression-free survival (Figure 3). Calibration slope was 0.944. In decision curve analysis, there was incremental net benefit compared to a strategy of ‘follow-all’ at risk thresholds ≥0.04 (Figure 4). At risk thresholds ≥0.26, net benefit using the model was inferior to a strategy of ‘follow-none’. This equates to net benefit for a clinician using the model who would investigate between 4.8 and 25 men to detect 1 progression event.

In sensitivity analysis, we excluded 7 men with imputed data for the final variables. Discrimination remained high (C-index 0.844, 95% CI 0.718–0.968). The calibration curve showed close agreement between predicted and observed progression-free survival, with a calibration slope of 1.177 (Figure S3). Sensitivity analysis excluding men with GG3 reclassification within 1 year was not possible owing to no men fulfilling this criterion.

## 4. Discussion

With a contemporary European multicentre cohort, we have developed a multivariable model for predicting the progression on AS using a clinically useful endpoint. This model demonstrated good discrimination, calibration and clinical utility for predictions up to 4 years post-diagnosis in both internal and external validation. Further external validation is now required in larger cohorts, followed by development of a risk calculator for clinical application.

Our model presents several potential advantages. First, our multicentre cohort increases power for model development but also improves generalisability by reflecting different AS eligibility criteria and practice. For example, the Lille and Valencia cohorts contained a greater proportion of GG1 disease than the Cambridge cohort (Table 2). Second, including men with GG2 disease is particularly important as many guidelines support AS for this group despite an inherently higher progression risk [20]. Despite the need to quantify risk in these men, recent models have only studied GG1 disease [21,22,23]. Third, we believe our choice of endpoint, ≥CPG3 progression, has better translation to clinical practice than using pathological upgrading. The latter is important for identifying men with stable GG1 disease who can safely continue AS. However, GG2 disease presents a wide spectrum of risk and upgrading from GG1 to GG2 does not necessarily require stopping AS, nor does it necessarily require pursuing treatment. In contrast, ≥CPG3 progression is a more pragmatic endpoint that more strongly warrants starting treatment, the use of which could reduce uncertainty and improve decision making [15]. Last, our model is amongst the first to incorporate MRI score, with the exclusion of this now near-ubiquitous diagnostic parameter a limitation cited with previous models [21,22].

The proposed use of our model lies at the time of diagnosis for men starting AS. Men with lower risk predictions could be candidates for lower-intensity surveillance [15]. Less frequent AS investigations could potentially improve adherence, safety, and costs. In contrast, more intense AS strategies could be better justified with higher risk predictions. Alternatively, higher risk predictions at diagnosis may inform discussions regarding pursuing primary treatment instead. The use of such a model to reduce investigations in lower-risk men could also save on costs. In addition, our model had very good discrimination in our development cohort when excluding men with GG1 that progressed to GG3 within 1 year. These cases likely represent the misclassification of a more aggressive tumour rather than the true progression of an indolent one [24,25,26]. With improving diagnostics and reduced sampling error, our model could perform better with more contemporary patients. Following further external validation, we propose the development of a web-based risk calculator to facilitate simple clinical application. We note that such an application would be easily constructable with R Shiny, as has been done previously [21,22,23]. We stress that our model does not seek to recommend specific follow-up plans, but instead to inform discussions on how best to monitor a given patient.

Our work has certain limitations. Although multicentre, our development cohort remained modestly sized and our external cohort small. Furthermore, there were differences between cohorts in AS eligibility criteria, practice, and available follow-up time (Table 2, Supplementary Text S1, Figure S1). This heterogeneity may subsequently have affected the final model’s performance. However, calibration remained good in external validation.

Third, our model is designed for use at diagnosis and does not provide dynamic predictions updatable with investigation results [21,22]. However, defining progression risk at diagnosis is arguably the most important time for informing discussions and follow-up strategy. Furthermore, our model performed well and is simple in only requiring diagnostic data.

Other limitations relate to our composite outcome. As with dichotomous endpoints, there is ambiguity regarding reaching this endpoint for men with diagnostic parameters already near the threshold. For example, a man with GG2 and PSA 9.9 ng/mL at diagnosis with subsequent surveillance PSAs of 10.0 ng/mL would be defined as ≥CPG3 progression. However, this arguably does not present true clinical progression and the variation in PSAs could be within laboratory error. In addition, radiological progression to T3–4 without pathological confirmation may not represent true progression. However, the specificity and negative predictive value of MRI for staging are known to be high [27].

We also had limitations pertaining to model variables. The men studied were predominantly Caucasian, and subsequently, we could not consider ethnicity as a model variable. This will be an important consideration when selecting further external validation cohorts. In addition, our MRI score variable here incorporated both PI-RADS and Likert systems, though we note there is comparable performance between the two [28,29]. MRI score was also missing in 18.2% of the development cohort. However, model performance remained good when men with imputed data were excluded. The inclusion of MRI score is additionally limited by interobserver variation and there was no central standardisation of imaging or biopsy reporting [30].

## 5. Conclusions

With a multicentre prospective cohort, we have successfully developed and validated a multivariable model for predicting progression to ≥CPG3 (unfavourable intermediate-risk) disease in men starting AS. Our model, which incorporates MRI data, demonstrated good discrimination, calibration, and clinical utility for predictions at 4 years post-diagnosis. The STRATCANS model could guide discussions and decision making between clinician and patient in deciding personalised AS strategies. Men with lower risk predictions could safely pursue a less-intense surveillance strategy, potentially improving adherence, safety and costs. Meanwhile, men with higher risk predictions could be offered more intensive AS strategies. Validation in larger cohorts is now required to evaluate our model further, followed by the development of a web application to facilitate clinical use.

## Figures and Tables

**Table 1:** Criteria for the Cambridge Prognostic Groups. GG, grade group; PSA, prostate-specific antigen.

Cambridge Prognostic Group	Criteria
1	GG1 **AND** PSA < 10 ng/mL **AND** Stage T1–2
2	GG1 **AND** PSA 10–20 ng/mL **AND** Stage T1–2 **OR** GG2 AND PSA < 10 ng/mL **AND** Stage T1–2
3	GG2 **AND** PSA 10–20 ng/mL **AND** Stage T1–2 **OR** GG3 **AND** PSA ≤ 20 ng/mL **AND** Stage T1–2
4	One of: GG4 **OR** PSA > 20 ng/mL **OR** Stage T3
5	**At least two of:** GG4 **OR** PSA > 20 ng/mL **OR** Stage T3 OR G5 **OR** Stage T4

**Table 2:** Summary cohort characteristics including diagnostic baseline parameters and surveillance outcomes, stratified by location. The multicentre model development cohort comprised men from Cambridge, Lille, and Valencia. The Cardiff cohort was used for external validation. CPG, Cambridge Prognostic Group; PI-RADS, Prostate Imaging-Reporting and Data System; PSA, prostate-specific antigen.

Variable	Cambridge *n* = 394 ^1^	Lille *n* = 176 ^1^	Valencia *n* = 313 ^1^	Cardiff *n* = 151 ^1^
**Age (years)**	66.9 (61.1, 71.1)	65.5 (60.3, 69.8)	64.8 (59.8, 69.5)	67.0 (61.0, 71.0)
**PSA (ng/mL)**	6.21 (4.51, 8.09)	6.29 (4.85, 8.70)	5.20 (3.89, 7.20)	6.30 (4.95, 7.95)
**Grade group**				
1	312 (79%)	173 (98%)	297 (95%)	130 (86%)
2	82 (21%)	3 (1.7%)	16 (5.1%)	21 (14%)
**CPG**				
1	258 (65%)	147 (84%)	270 (86%)	113 (75%)
2	136 (35%)	29 (16%)	43 (14%)	38 (25%)
**Prostate volume (mL)**	47.0 (33.0, 69.6)	55.0 (40.0, 75.5)	44.0 (32.8, 60.5)	49.0 (35.0, 65.0)
Unknown	10	1	1	2
**MRI score**				
1	90 (33%)	66 (38%)	118 (43%)	46 (32%)
2	10 (3.7%)	0 (0%)	49 (18%)	3 (2.1%)
3	52 (19%)	43 (25%)	55 (20%)	66 (46%)
4	74 (27%)	61 (35%)	43 (16%)	16 (11%)
5	47 (17%)	2 (1.2%)	12 (4.3%)	14 (9.7%)
MRI not performed	121	4	36	6
**Positive family history**	67 (21%)	35 (20%)	56 (18%)	29 (19%)
Unknown	82	2	10	0
**Core positivity**	0.14 (0.08, 0.25)	0.08 (0.08, 0.15)	0.08 (0.05, 0.11)	0.20 (0.10, 0.30)
Unknown	36	2	10	3
**Reached ≥ CPG3 progression**	57 (14.5%)	11 (6.3%)	38 (12.1%)	11 (7.3%)
**Time-to-progression (years)**	1.9 (1.0, 3.5)	2.5 (1.1, 3.4)	1.1 (0.6, 2.6)	2.0 (1.2, 3.3)
**Follow-up of censored men (years)**	3.6 (2.0, 5.6)	3.1 (1.7, 4.7)	2.4 (0.8, 3.6)	3.8 (3.0, 4.4)

^1^ Median (IQR); n (%).

## Data Availability

Datasets are available from the corresponding author upon reasonable request and agreement from other co-authors.

## Supplementary Materials

The following supporting information can be downloaded at: www.mdpi.com/article/10.3390/jcm12010216/s1, Figure S1: Box plots demonstrating follow-up time for censored men (A) and time-to-progression (B), stratified by site; Figure S2: Kaplan–Meier curves for each centre’s cohort studying AS discontinuation-free survival (any reason). These are shown for all men included (A), and specifically for men who did not reach ≥CPG3 progression during study follow-up (B); Figure S3: Calibration curves at 4 years in a sensitivity analysis where men with imputed data were excluded for A, internal validation, and B, external validation. Calibration slope was 1.0008 in internal validation and 0.829 in external validation; Figure S4: Calibration plot for model performance at 4 years in t internal validation in a sensitivity analysis where men with GG1 disease at diagnosis, but who had GG3 disease on re-biopsy within a year, were excluded. Table S1: The number of men in each cohort who reached ≥CPG3 progression according to individual definitions of this composite endpoint; Table S2: Unadjusted associations (univariable Cox proportional hazards regression) and adjusted associations (multivariable Cox proportional hazards regression) between candidate predictor variables and the outcome in the development cohort.
