# Supplementary material for: Development and External Validation of the STRATified CANcer Surveillance (STRATCANS) Multivariable Model for Predicting Progression in Men with Newly Diagnosed Prostate Cancer Starting Active Surveillance
Source: J Clin Med. 2022 Dec 27;12(1):216. doi: 10.3390/jcm12010216 (PMC9821695; doi:10.3390/jcm12010216)
Supplement: Supplementary file 1 [file jcm-12-00216-s001.zip › jcm-1912391-supplementary.pdf]

Supplemental Material

Text S1: Centre-specific active surveillance eligibility criteria and follow-up protocols

Cambridge

In the early years of this programme, MRI was mainly used after diagnosis and, if needed, to guide repeat biopsy if samples on MRI were not concordant. For later years, men had pre-biopsy MRI to guide diagnostic biopsy sampling. All had 3-monthly PSA testing and annual repeat MRI [Thurtle et al., 2018; doi: 10.1111/bju.14166]. Protocol interval re-biopsies were scheduled at 12 months and 3 years. 3 consecutive rises in PSA or change in mpMRI result triggered an earlier targeted image-fusion re-biopsy. Data collection were approved by Institutional Review Board approval (Cambridge University Hospitals NHS Foundation Trust, Cambridge, UK; registration number: 3592).

Lille

PSA was not an exclusion criteria. All had pre-biopsy MRI followed by targeted biopsy if a lesion was visible (PI-RADS 3-5). If targeted biopsies of this suspicious lesion were then negative for cancer, the patient was then considered for AS. Men with non-suspicious MRIs (PI-RADS 1-2), these men were also considered. Follow-up comprised 6-monthly PSA, and yearly digital rectal exam. If there were two instances of PSA velocity >0.5ng/mL/year, MRI was repeated followed by repeat biopsy if a suspicious lesion was visible. This 'for cause only biopsy' strategy was started in 2013 based on previous analyses [Olivier et al., 2019; doi: 10.1007/s00345-018-2420-6]. Follow-up comprised 6-monthly PSA, and yearly digital rectal examination. There were no pre-established frequency for MRI or biopsies. If there were a PSA velocity >0.5 ng/mL/year, MRI was repeated. A repeat biopsy was then performed if a suspicious lesion was observed. If no suspicious lesion was seen, a systematic biopsy was performed if PSA continued to rise. Database protection authorisation and patient consent was obtained as requested by the local ethical committee.

Valencia

GG2 was only permitted in men aged ≥70 years. Furthermore, if prostate volume was >60mL with PSA >10 ng/mL, but PSA density <0.20 ng/mL^2, these men were also eligible for AS. were required. A baseline MRI was performed at AS entry or before a repeat confirmatory biopsy during the first year of inclusion, if the diagnostic biopsy was a non-image guided 10–12 cores TRUS-guided biopsy. AS was only used if the image-guided confirmatory biopsy continued to meet the inclusion criteria. During follow-up, PSA and digital rectal examination were performed every 6 and 12 months, respectively. Biopsies were repeated every 2-3 years, always preceded by MRI. Both transrectal and transperineal approaches were used depending on prostate size and location of suspicious lesion, if MRI-visible. Data were collected with signed written consent under the CAPROSIVO study, accepted by the local ethics committee.

Cardiff

Men eligible for AS at this centre had low risk disease according to the UK National Institute for Health and Care Excellence (NICE; PSA <10 ng/mL, GG1, T1-T2a). Men with intermediate risk cancer (PSA 10-20 ng/mL, GG2, stage T2b) were eligible only

if there was <10% of Gleason pattern 4 and small volume disease on TRUS biopsy, with <50% core involvement in ≤3 cores. Follow-up was performed according to NICE guidelines at the time [https://www.nice.org.uk/guidance/ng131/evidence/full-guideline-pdf-6781033550]. During year 1 of AS, PSA should be measured every 3-4 months, digital rectal examination every 6-12 months, and re-biopsy at 12 months. In years 2-4, PSA was measured every 3-6 months, and digital rectal examination again every 6-12 months. From year 5 of active surveillance and onwards, PSA was measured every 6 months, and digital rectal examination repeated every 12 months. If there is concern about clinical or PSA changes at any time during AS, men would undergo repeat MRI with or without re-biopsy.

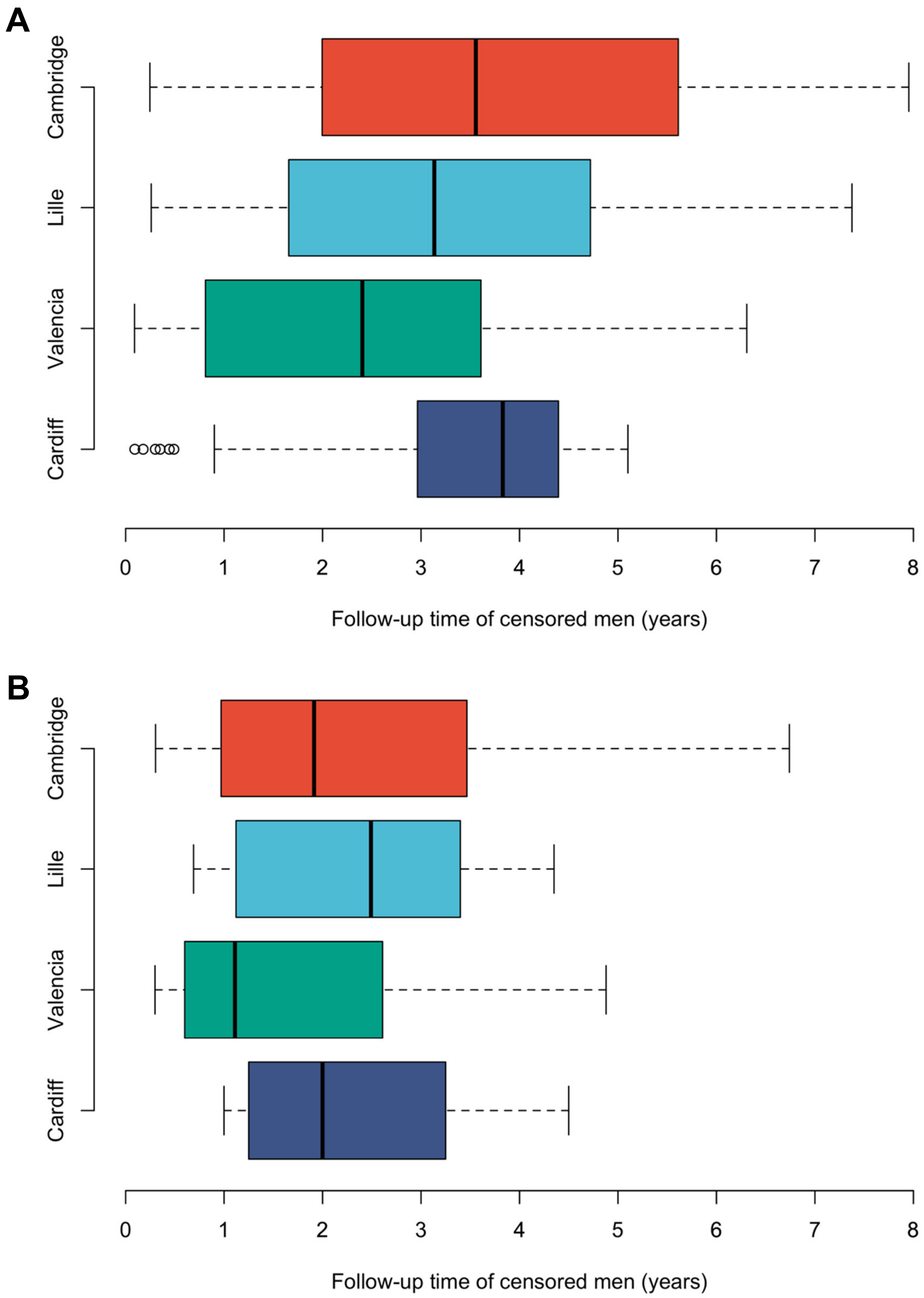

Figure S1: Box plots demonstrating follow-up time for censored men (**A**) and time-to-progression (**B**), stratified by site.

**Definition of ≥CPG3 progression**	**Cambridge** *n = 56*	**Lille** *n = 11*	**Valencia** *n = 38*	**Cardiff** *n = 11*
Upgrading to ≥GG3	16 (28.1%)	3 (27.3%)	27 (71.1%)	3 (27.3%)
GG2 with PSA ≥10 ng/mL	6 (10.5%)	4 (36.4%)	5 (13.2%)	8 (72.7%)
Radiological progression to T3-4	12 (21.1%)	3 (27.3%)	3 (7.9%)	0
PSA >20 ng/mL	23 (40.4%)	1 (9.1%)	4 (10.5%)	0

Table S1: The number of men in each cohort who reached ≥CPG3 progression according to individual definitions of this composite endpoint.

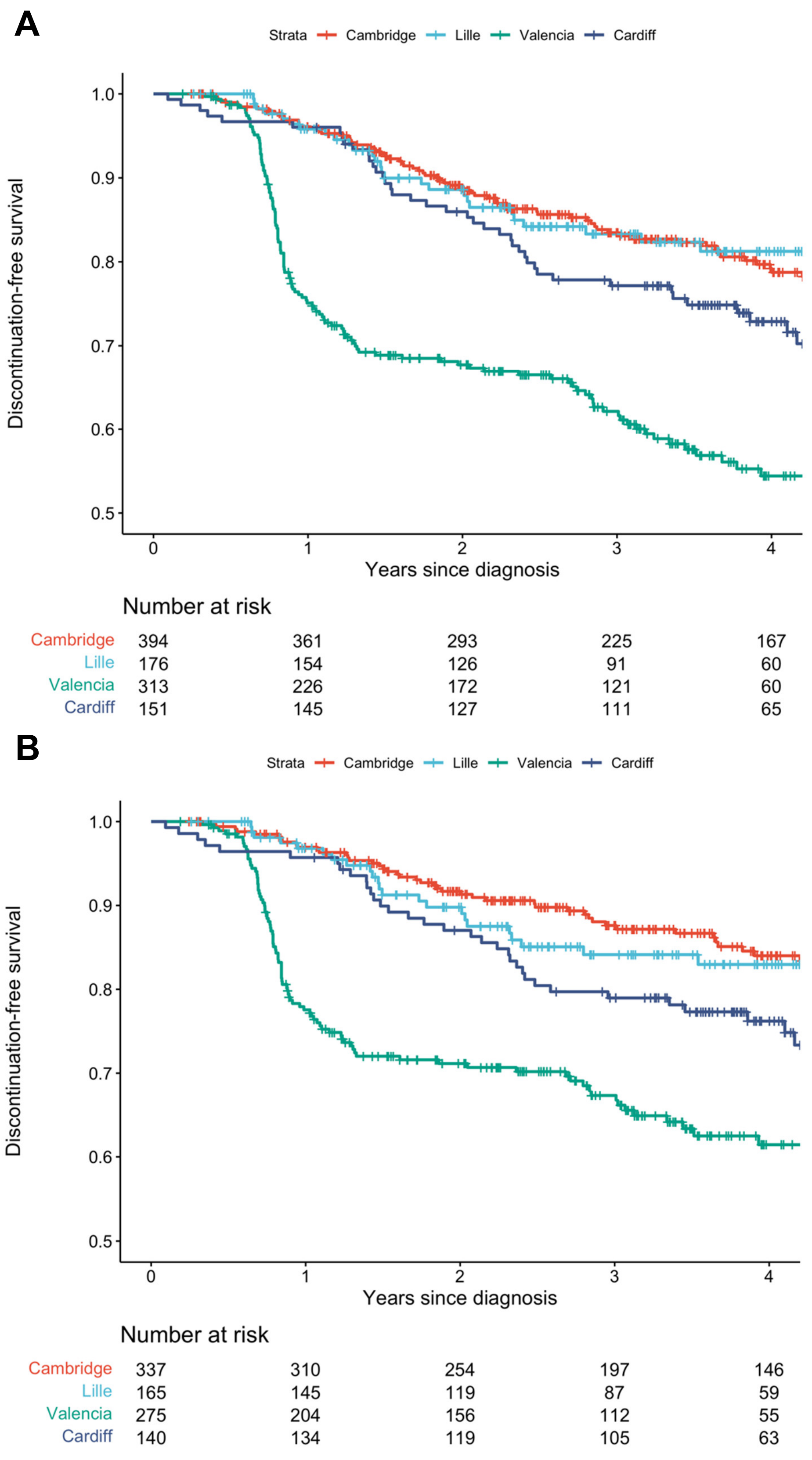

Figure S2: Kaplan-Meier curves for each centre's cohort studying AS discontinuation-free survival (any reason). These are shown for all men included (**A**), and specifically for men who did not reach ≥CPG3 progression during study follow-up (**B**).

	Unadjusted			Adjusted		
**Variable**	**HR**[1]	**95% CI**[2]	***p***	**HR**[1]	**95% CI**[2]	***p***
PSA	1.14	1.08, 1.19	<0.001	1.20	1.13, 1.27	<0.001
Prostate volume	1.00	0.99, 1.00	0.5	0.990	0.982, 0.998	0.01
Grade Group 2	4.19	2.73, 6.44	<0.001	4.16	2.64, 6.54	<0.001
MRI score 4-5	2.33	1.53, 3.55	<0.001	1.85	1.21, 2.84	0.005
Age	1.04	1.02, 1.07	0.003	-	-	-
Core positivity	8.27	2.59, 26.39	0.004	-	-	-
Positive family history	0.87	0.26, 2.87	0.6	-	-	-

[1] HR = Hazard Ratio
[2] CI = Confidence Interval

Table S2: Unadjusted associations (univariable Cox proportional hazards regression) and adjusted associations (multivariable Cox proportional hazards regression) between candidate predictor variables and the outcome in the development cohort.

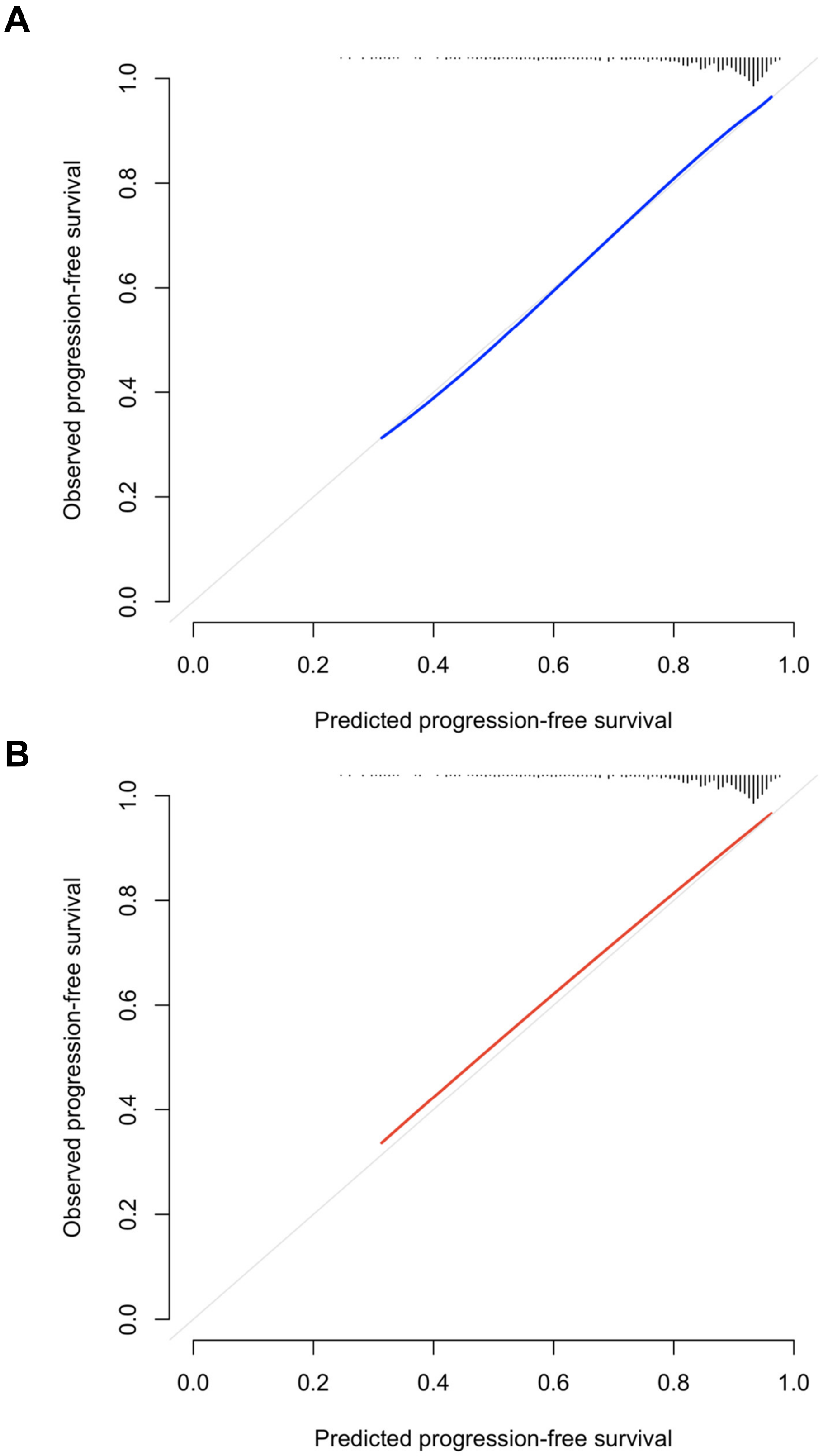

Figure S3: Calibration curves at 4 years in a sensitivity analysis where men with imputed data were excluded for **A**, internal validation, and **B**, external validation. Calibration slope was 1.0008 in internal validation and 0.829 in external validation.

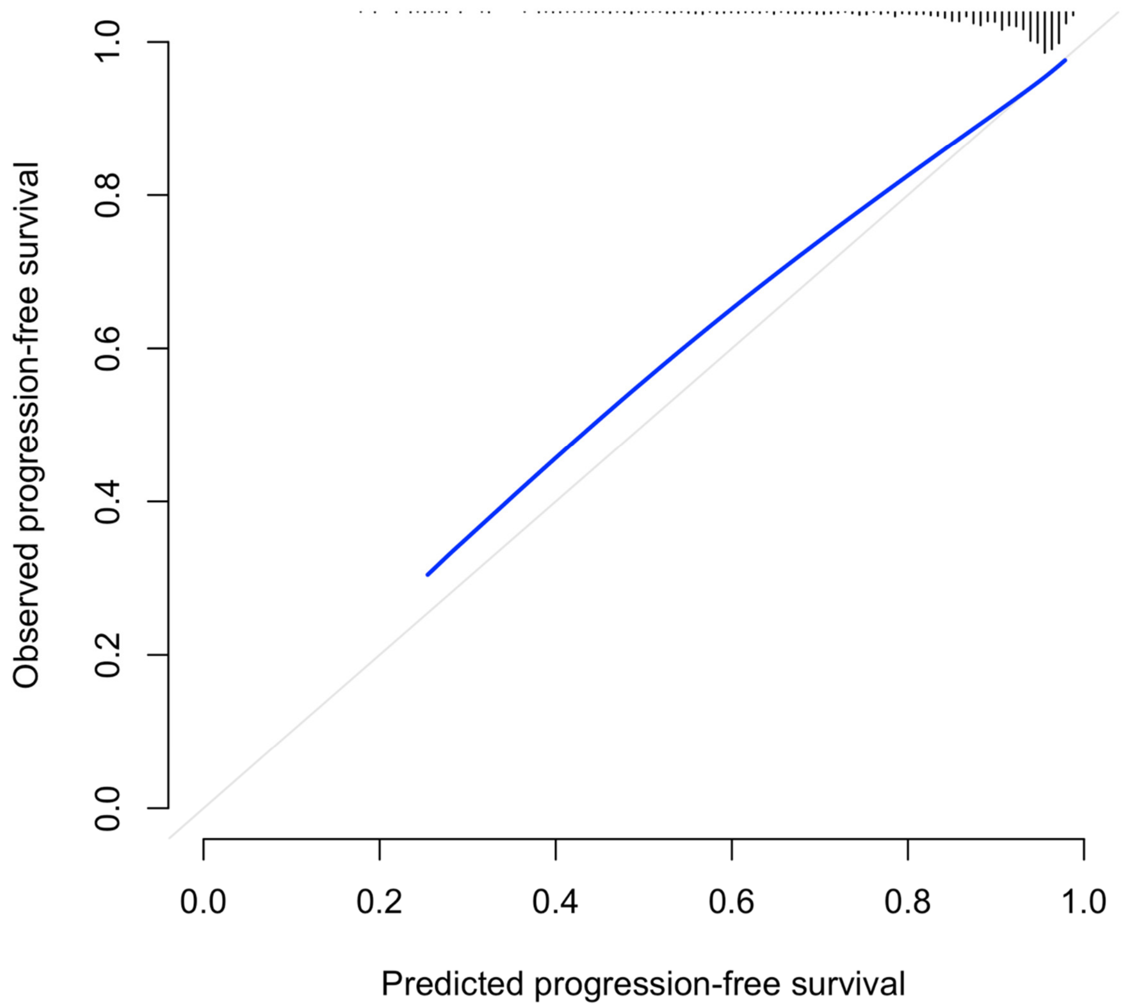

Figure S4: Calibration plot for model performance at 4 years in t internal validation in a sensitivity analysis where men with GG1 disease at diagnosis, but who had GG3 disease on re-biopsy within a year, were excluded.
